# Evaluation of Clinical Efficacy of Biodegradable Chip Containing *Salvadora persica* (miswak) Extract in Chitosan Base as an Adjunct to Scaling and Root Planing in the Management of Periodontitis

**DOI:** 10.18295/squmj.6.2024.028

**Published:** 2024-08-29

**Authors:** Fouad H. Al-Bayaty, Azwin A. Kamaruddin, Mohd A. Ismail, Mazen M.J. Al-Obaidi

**Affiliations:** 1 Faculty of Dentistry, Department of Periodontology, MAHSA University, Selangor, Malaysia; 2 Faculty of Dentistry, Department of Comprehensive Care, Universiti Teknologi MARA, Selangor, Malaysia; 3 Klinik Pergigian Merlimau (Principal), Melaka, Ministry of Health, Malaysia; 4 Science Department, College of education, University of Technology and Applied Sciences, Rustaq, Oman

**Keywords:** Chitosan, Biocompatible Materials, Isothiocyanates, Salvadora, Periodontitis

## Abstract

**Objectives:**

This study attempted to develop 2 biodegradable periodontal chips containing *Salvadora persica* (miswak) or benzyl isothiocyanate (BITC) extracts and evaluate their clinical effectiveness in managing periodontitis.

**Methods:**

This clinical trial was conducted at the Faculty of Dentistry, Universiti Teknologi MARA Shah Alam, Selangor, Malaysia, from September 2010 to April 2012. Periodontal chips were formulated using *S. persica*, benzyl isothiocyanate (BITC) and chitosan extracts. All patients were treated with full mouth scaling and root planing at baseline. Thereafter, the periodontal pockets (≥5 mm in length) were divided into 4 groups: the control group; group 2 (plain chitosan chip); group 3 (*S. persica* extract); and group 4 (BITC extract). Plaque index (PI), bleeding on probing (BOP), periodontal probing pocket depth and clinical attachment levels were recorded at days 0 and 60 only.

**Results:**

A total of 12 patients participated in this study. Overall, 240 periodontal pockets were evaluated. The study revealed significant improvements in PI, BOP and reduction in periodontal pocket depth in all 4 groups (*P* <0.05). The improvement in clinical attachment level was significantly higher (*P* <0.001) among the group that received *S. persica* chips compared to the control and other chip-treated groups.

**Conclusion:**

Periodontal chips containing *S. persica* can be used as adjuncts to treat patients with periodontitis.

**Advances in Knowledge**

*- The use of Salvadora persica (miswak)-based periodontal chips facilitates a significant decrease in bleeding on probing, plaque index and periodontal pocket depth. Furthermore, there is a more significant rise in clinical attachment level compared to chips created using other extracts*.
*- S. persica-based periodontal chips’ usage can serve as a beneficial additional method of treating patients with periodontitis, especially during follow-up involving maintenance. This study’s findings support the efficacy of S. persica-based therapies in enhancing periodontal health and justifies the need for more investigation into their therapeutic capabilities regarding periodontal care*.

**Application to Patient Care**

*- This study showed that S. persica reduces plaque, improves gingival health and promotes wound healing*.
*- The biodegradable chip, containing thymoquinone, showed promising results with respect to reducing plaque, improving gingival health and promoting wound healing. Thus, S. persica-based periodontal chips are suggested as a viable and economical alternative to other chips in the management of periodontal disorders*.

Periodontitis is an inflammatory condition that affects the periodontium (the supporting structure of the teeth). Periodontitis extends beyond the gingiva and causes damage to the connective tissue that holds human teeth in place. The bacteria found in dental plaque are commonly acknowledged as the primary cause of inflammatory periodontal diseases.^1^^,^^2^ Traditionally, periodontal therapy aims to modify the periodontal environment, creating conditions that facilitate the removal of dental plaque.

Treatment routines for periodontitis include: providing oral health guidance to achieve sufficient oral hygiene, performing scaling to remove plaque and tartar, correcting any faulty dental restorations, performing root planing to smooth the tooth roots and surgically addressing pockets or anatomical flaws.^3^ Nevertheless, as knowledge of the bacterial causes of periodontal disease have advanced, the use of antibacterial medicines has become a crucial component of periodontal treatment.^4^ Moreover, the care of periodontitis presently involves preventing the further decline of periodontal support by eradicating certain pathogenic bacteria present in the periodontal pocket. This can be achieved through the process of mechanical scaling and root planing (SRP). However, the depth of the periodontal pockets can lead to significant differences in the efficiency of SRP. Thus, antimicrobial medicines should be used in conjunction with these procedures to improve periodontitis management.^5^ Antimicrobial drugs can be transported to the periodontitis-affected areas through either systemic or local application.^6^ Indeed, the periodontal pockets serve as a natural liquid enclosure filled with gingival crevicular fluid, facilitating convenient access for delivery devices. The gingival crevicular fluid serves as a vehicle through which the medicine can be distributed throughout the periodontal pocket. Therefore, the periodontal pocket is an ideal location for the administration of medication via a localised sustained-release delivery system.

PerioChip® (Dexcel Pharma, Jerusalem, Israel) is an innovative biodegradable delivery system designed to reduce pocket depth in cases of chronic periodontitis. It is usually used as an adjunct treatment with SRP.^7^ The chip is a compact, orange-tan, rectangular object with one rounded end, designed to be inserted into periodontal pockets. Each PerioChip® (Dexcel Pharma) weighs approximately 7.4 mg and contains 2.5 mg of chlorhexidine gluconate, which is an antimicrobial agent. This agent is held in a biodegradable matrix made of hydrolysed gelatin that is cross-linked with glutaraldehyde.

Multiple studies have demonstrated the clinical efficacy of dental practitioners and their patients in effectively managing periodontitis over an extended period of time.^8^–^10^ Nevertheless, the utilisation of PerioChip® (Dexcel Pharma) has demonstrated very few negative consequences in previous studies. This may be attributed to its primary constituent, namely chlorhexidine gluconate. Such an assumption is substantiated by the following observations: brown discoloration of teeth, certain restorative materials and mucosa, as well as the presence of a bitter taste in the mouth. Additionally, in some cases, the use of chlorhexidine in mouth-rinses has been associated with the sloughing of oral mucosa.^11^–^13^ In order to mitigate these detrimental effects, numerous researchers are currently exploring the potential utilisation of natural products, such as plant extracts and herbs, as viable alternatives to PerioChip® (Dexcel Pharma). In this light, previous studies have already examined the comparable efficacy of plant extracts and herbs such as *Myrtus communis*, *Arnica montana* and *Hamamelis virginiana* against periodontopathic bacteria, revealing their potential antibacterial activity.^14^–^16^

The current study investigates the concomitant efficacy of *Salvadora persica*, an evergreen tree with medicinal properties that has been utilised for over 10 centuries by various populations, particularly Islamic communities residing in Middle East, India and Africa. Many products have been developed from this medicinal tree, such as toothbrushes made from the roots and small branches of this tree. Miswak (sewak, siwak) is a chewing stick obtained from the roots of the *S. persica* tree, otherwise known as the Arak tree or Peelu tree. According to the literature, *S. persica* contains substances that have plaque-inhibiting properties and antibacterial effects against various cariogenic bacteria commonly present in the oral cavity.^16^ The therapeutic applications of *S. persica* have led to its inclusion in dental hygiene products such as toothpaste, mouth-rinses and endodontic irrigation solutions.^17^ Moreover, the steam-distillable oil derived from the root of *S. persica* consists of 10% benzyl nitrate and 90% benzyl isothiocyanate (BITC).^17^ Thus, *S. persica* exhibits a diverse array of bactericidal properties.^16^–^19^

On the other hand, BITC is a naturally occurring compound found in plant tissues. It is present in Indian cress (*Tropaeolum majus* L.) and garden cress (*Lepidium sativum* L.); moreover, significant quantities of BITC can be found in cruciferous vegetables such as cabbages, brussels sprouts, cauliflower and broccoli.^20^

Chitosan has gained attention as a carrier in medication delivery procedures because of the following properties: stability, ability to break down naturally, lack of toxicity, impressive adherence to mucus and improvement of permeability.^13^^,^^21^

Currently, there is a lack of research on the potential development of a periodontal chip that incorporates *S. persica* to treat chronic periodontitis. Hence, this study aimed to develop a biodegradable periodontal chip that incorporates *S. persica* and assess this chip in a chitosan base as a targeted drug delivery system designed to treat periodontitis.

## Methods

This randomised, single-blinded clinical trial was conducted at the Faculty of Dentistry, Universiti Teknologi MARA Shah Alam, Selangor, Malaysia, from September 2010 to April 2012. This study consisted of 2 parts: a laboratory process and a clinical trial. The laboratory process included the manufacture of periodontal chips containing thymoquinone, while the clinical trial involved the implementation of periodontal therapy and the insertion of the chips.

All participants had at least 4 non-adjacent teeth with periodontal pockets measuring ≥5 mm. Patients with a medical history of systemic disease that could potentially affect the progression of periodontal disease or necessitate prophylactic antibiotics prior to dental treatment, recent use of antibiotics or any form of periodontal treatment within the preceding 3 months, the presence of overhanging restorations, pregnancy, smoking habits and allergy to *S. persica* were excluded.

The *S. persica* sticks (miswak) utilised in this study were purchased from a local store in Malaysia. The material was subsequently crushed into a nanoparticle powder form using a Hammer Mill blender (AVEKA Inc, Woodbury, Minnesota, USA). The particle size of the powder was analysed using the Master sizer 2000 instrument (Malvern Panalytical, Malvern, UK) for the purpose of verifying the particle size. The powder was subsequently extracted using ethanol. In total, 200 gm of *S. persica* powder resulted in a yield of 15 g of dry extraction.

A solution containing 1% acetic acid was prepared by adding it to 2.5 g chitosan powder (Sigma-Aldrich, St. Louis, USA). The mixture was allowed to stand overnight. Subsequently, it was dissolved in water and subjected to sonication to achieve a uniform mixture. This mixture was inserted in specially designed rectangular glass moulds that were lined with aluminium foil. After being left to dry overnight at room temperature, the resulting layer was divided into small rectangular chips (0.5 × 0.5 cm) with a thickness of 0.16 ± 0.02 mm. Subsequently, the chips were enveloped with aluminium foil and stored in aseptic vials at ambient temperature.^22^

The *S. persica* (2.5 mg, 100% w/w) were fragmented and mixed with chitosan that had been steeped in 1% acetic acid overnight. Both components were subjected to sonication (so that the authors could produce a uniform combination) and then transferred to a specially designed rectangular glass mould that was lined with aluminium foil. Following an overnight period where the mould was left to dry at room temperature, the resulting layer was divided into small rectangular chips (0.5 × 0.5 cm) with a thickness of 0.16 ± 0.02 mm A content uniformity test was conducted on a few randomly selected chips to confirm the precise amount of medication delivered in each chip. The chips were then transferred into aseptic vials and stored at an ambient temperature. The identical protocols were replicated using 0.25 mg of BITC (Sigma-Aldrich).

In this trial, a ‘vial’ method was utilised for the *in vitro* release study. A total of 10 chips made using *S. persica* were inserted into glass vials. Each vial contained 5 mm of phosphate buffer saline. At 2–6-hour intervals, 1.0 mL samples were periodically taken from the vials. Additionally, samples were taken at 1^st^, 2^nd^, 3^rd^, 5^th^, 7^th^, 9^th^, 11^th^ and 15^th^ day of the trial. On each occasion, a given sample was replaced with fresh phosphate buffer saline to ensure the adequate availability of media for a proper breakdown. The samples were examined using a spectrophotometer set at a wavelength of 350 nm. The concentrations of *S. persica* in the samples were determined using the calibration curve established in the phosphate buffer saline. An *in vitro* release was constructed from the obtained data.^22^

**Figure 1:**
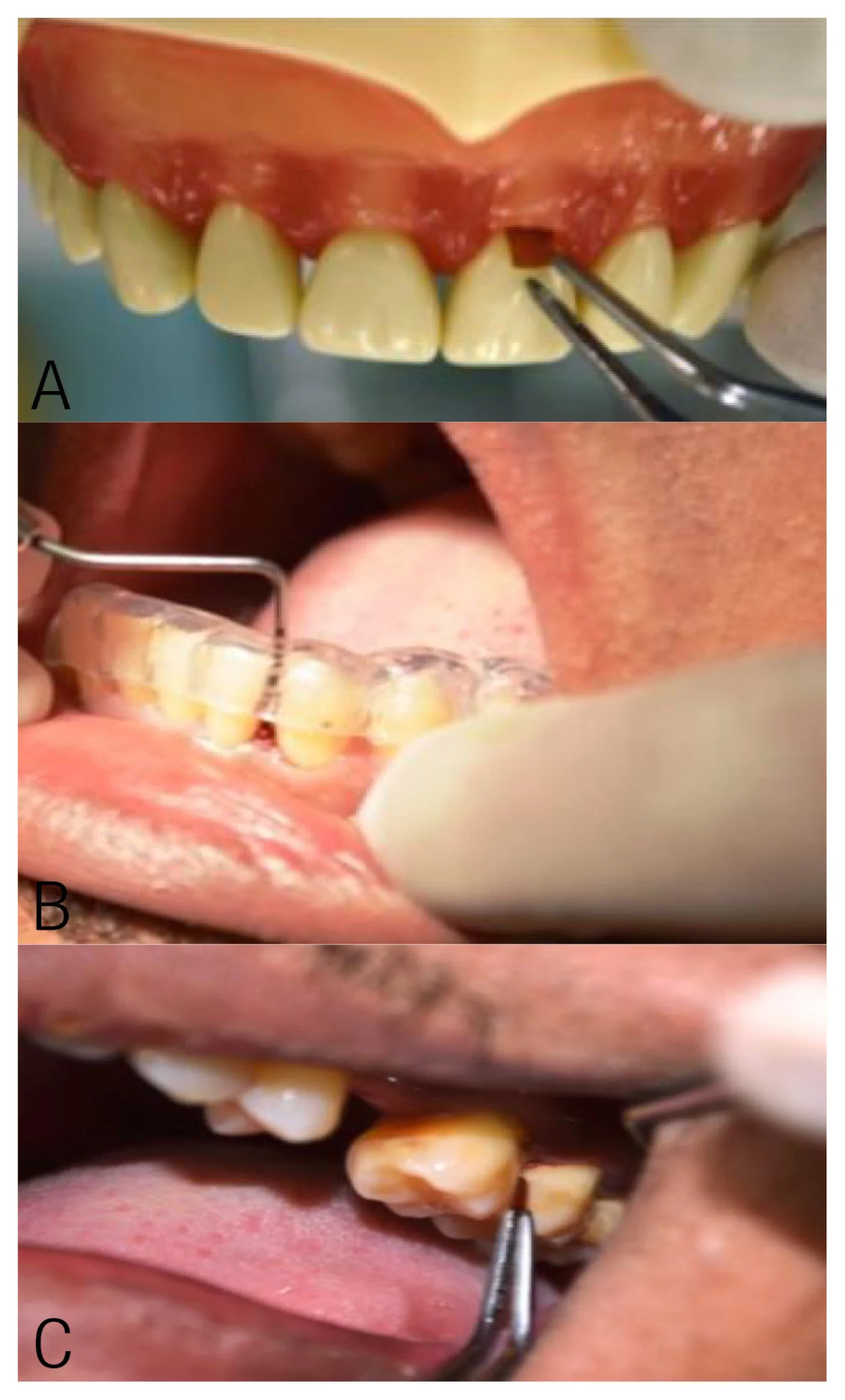
**A**: Photograph showing the insertion of the *S. persica* chip into the pocket on a Frasaco model for patient dental education, demonstration and simulation. **B**: Photograph of a patient’s mouth showing the method of measuring periodontal pocket using acrylic stent. **C**: Insertion of a *S. persica* chip inside the patient’s periodontal pocket of a patient.

Before the commencement of the clinical trial, the selected patients were given a subject information sheet that explained the research procedures in detail, including information on using the training model to educate them on how the chips should be inserted [Figure 1A]. An alginate impression of both dental arches of each patient was taken and a soft, transparent acrylic stent was constructed. The acrylic stent was used to precisely identify the specific location and ensure consistent measurements would be taken upon each visit [Figure 1B].

Initially, the examination involved a comprehensive assessment of the periodontal condition. All patients underwent full mouth scaling and polishing. They were also given instructions to follow a normal and effective oral hygiene regimen that included brushing. A sole examiner (MAI), who was blinded to the administered therapies administered, conducted all the clinical measurements. All clinical parameters were measured on day 0 (i.e. pre-treatment) and day 60 post-treatment.

This study assessed each patients’ plaque index (PI), bleeding on probing (BOP), and probing pocket depth (PPD) using a University of North Carolina Probe periodontal probe; the presence or absence of BOP was categorised in terms of a value of 0 or 1, respectively.^23^^,^^24^ BOP received a favourable rating if bleeding manifested within 20 seconds following pocket probing.

Following the collection of baseline measures, all study pockets underwent root planing with Gracey curettes (Hu-Friedy, Chicago, IL, USA) under local anaesthesia. This procedure was performed by AAK.

The chips were administered inside the patients’ periodontal pockets following SRP in groups 2, 3 and 4 [Figure 1C]. Prior to baseline, the 240 periodontal pockets were randomised into 4 groups with each group containing 60 sites: group 1 (control group) received SRP alone; group 2 received SRP with chitosan chip insertion; group 3 received SRP followed by *S. persica* chip insertion; and group 4 received SRP followed by insertion of the chip containing BITC.

Patients underwent examination 48 hours after the insertion of chips for evaluation. The patients were advised to refrain from using dental floss, mouth rinses or oral irrigation devices for a duration of 10 days after the insertion to prevent any movement of the chip throughout the study period. On day 14, the patients were recalled for a second chip insertion and their PI and BOP were checked. All clinical parameters were re-recorded on the last day of the clinical trial (day 60). Moreover, clinical attachment level (CAL) was measured by comparing each patients’ PPD before and after treatment. A reduction in the PPD indicated a gain in CAL and an increase denoted the worsening of PPD. CAL was measured by subtracting the distance between the cementoenamel junction and the free gingival margin from the PPD value.^25^

The PI, BOP and PPD measurements were used to calibrate the examiner internally. In a single patient, a total of 180 sites were assessed and the relevant data were documented. After 2 hours, the examiner proceeded to re-measure the 180 pockets. These measurements were repeated twice on the same patient. Thereafter, the relevant data were input into Statistical Package for Social Sciences (SPSS), Version 28.0 (IBM Corp., Armonk, New York, USA) and Cohen’s kappa coefficient was calculated. The analysis revealed a Kappa value of 0.81 (*P* <0.001), indicating an almost perfect agreement.

The mean values (per patient) of the clinical parameters were determined for each treatment group. Updates in the clinical parameters were computed for each site in both the test and control groups. Updates in the treatment groups’ PI, BOP, PPD and CAL between baseline and day 60 were analysed. SPSS (IBM Corp.) was used for data analysis. The statistical significance of differences was tested using a paired sample t-test, a Chi-squared analysis and a one-way analysis of variance. Statistical significance was set at *P* <0.05.

This study was approved by the Universiti Tecknologi MARA (600-RMI [5/1/6/01]). All participants providing written informed consent to participate in this research endeavour. The trial was registered in an international database (Current Controlled Trials Limited; registration number: ISRCTN29742423.

## Results

A total of 12 male patients diagnosed with periodontitis with a mean age of 41.8 ± 5.6 years (age range: 35–56 years), were selected to participate in this randomised clinical trial. A total of 1,656 periodontal pockets were assessed, however, only 240 periodontal pockets were analysed as they had a measurement of ≥5 mm.

**Figure 2:**
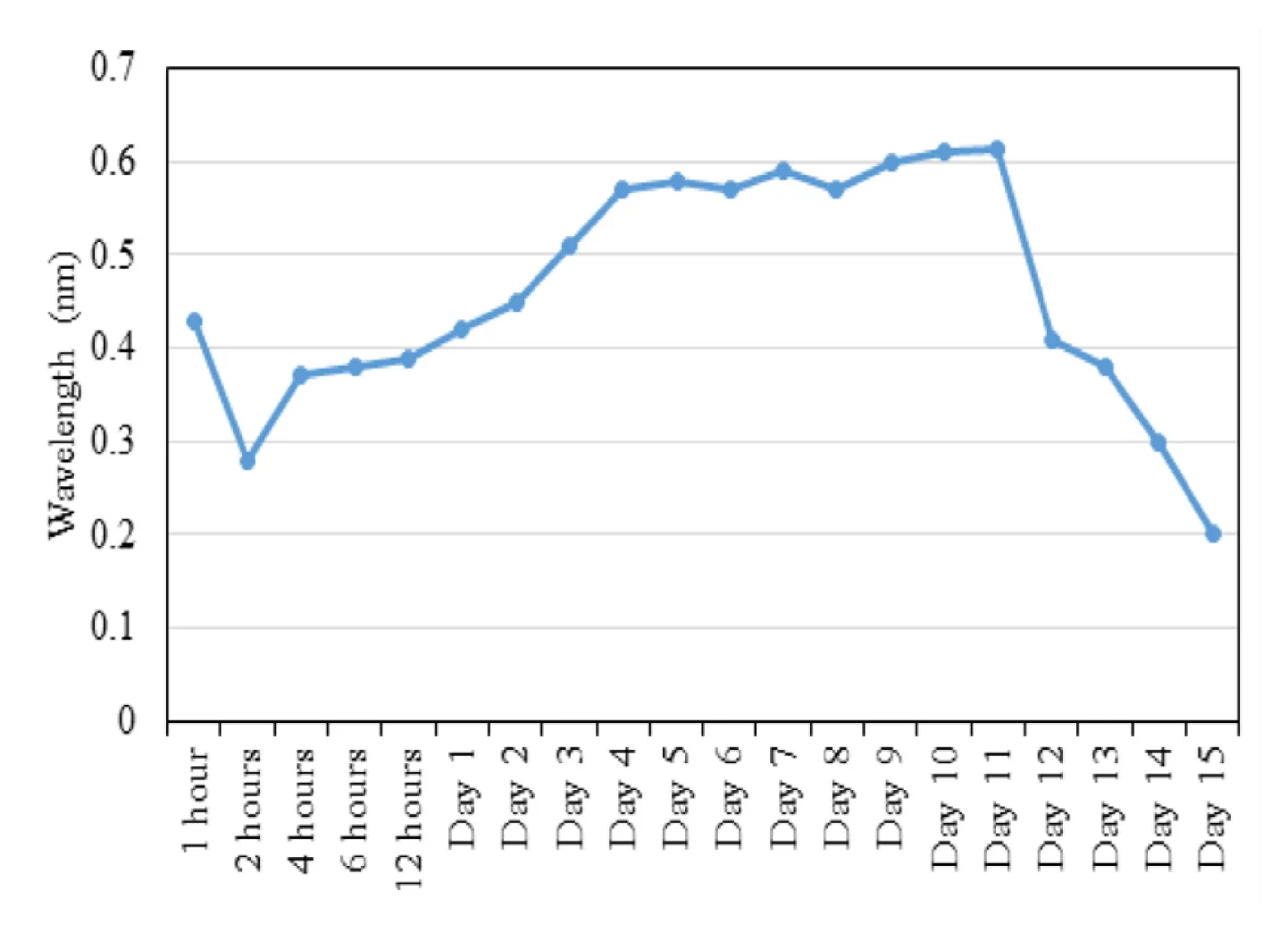
Results on the in vitro release of drugs by *S. persica*.

An *in vitro* release study, done over the span of 15 days, showed that from day 11, the discharge from the *S. persica* chip steadily decreased until day 15. At the end of this timeframe, there was a complete release of drugs (100%) [Figure 2]. This finding informed the rationale behind the decision to re-insert the periodontal chip after the 15^th^ day.

Each of the 4 study groups showed a decrease and enhancement in the number of sites with evident supragingival PI both before and after the therapy. The groups treated with *S. persica* (*P* = 0.026), BITC (*P* = 0.009) and chitosan (*P* = 0.031) chips all exhibited a notable enhancement in PI after the therapy. However, the PI of the control group did not show a significant change before and after treatment (*P* =0.292).

There was a notable improvement in BOP in all groups after the therapy. The improvements in BOP were found to be somewhat uniform across the control (*P* = 0.024), chitosan (*P* = 0.008), *S. persica* (*P* = 0.024) and BITC (*P* = 0.009) group after 2 months.

For each group, the average periodontal pocket was measured both before and after the treatment. The findings demonstrated statistically significant reductions in mean PPD after SRP across all 4 groups (*P* = 0.001 each). After 2 months, the PPD in the group treated solely with SRP decreased from 5.52 mm to 4.52 mm (1 mm reduction). Meanwhile, the PPD of the group that received SRP combined with chitosan reduced from 6.08 mm to 5.27 mm (0.81 mm reduction). Similarly, the group that received SRP and the *S. persica* chip group showed a PPD reduction from 7.15 mm to 5.60 mm (1.55 mm reduction). The group that received SRP combined with the BITC chip had a PPD reduction from 5.90 mm to 4.63 mm (1.27 mm reduction). The group that received SRP plus the *S. persica* chip demonstrated more noticeable improvements in PPD in comparison to the other groups [Table 1].

**Figure 3:**
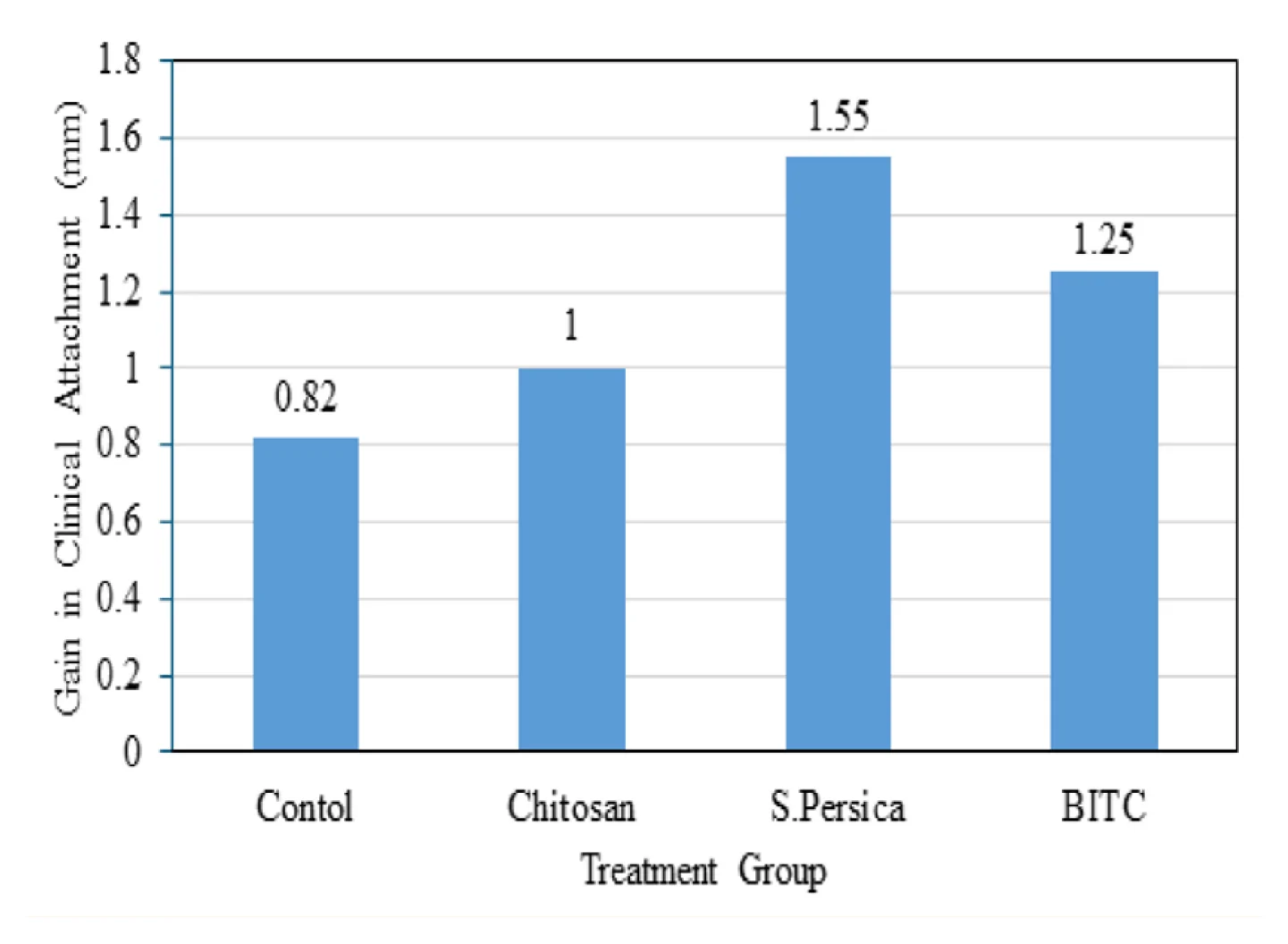
Comparative analysis of clinical attachment levels gain in 4 groups measured in mm.

An increase in CAL was found in all 4 groups. The group treated with *S. persica* demonstrated a notably greater improvement in CAL, exhibiting the highest gain of 1.55 mm, followed by the BITC group (1.25 mm), the chitosan group (1.00 mm) and the control group (0.82 mm) [Figure 3].

## Discussion

This study aimed to develop and assess the efficacy of a biodegradable chip containing an extract of *S. persica* in a chitosan base. This chip can be used as a targeted medication delivery system designed to treat periodontitis. The roots of the *S. persica* demonstrably possess an antimicrobial effect.^11^ Importantly, BITC is the primary antibacterial component found in *S. persica* extracts.

*S. persica* extracts and commercially synthesised BITC reportedly have a rapid and robust bactericidal effect on oral pathogens implicated in periodontal disease as well as various Gram-negative bacteria.^17^ Moreover, *S. persica* is reportedly efficacious as an anti-inflammatory and antioxidant agent, thus exhibiting therapeutic properties.^26^ Specifically, it modifies the structure of nitric oxide synthase isoforms and reduces the levels of pro-inflammatory cytokines such as IL-1, IL-6, IL-8, TNF and IFN.^27^^,^^28^ Additionally, it enhances the anti-inflammatory and antioxidant effects at the site of inflammation.^17^ These characteristics prove that *S. persica* extracts may have an important role to play in the management and progression of periodontal disease.

Previous studies have demonstrated that including a biodegradable chlorhexidine chip as an adjunct to treatment results in significant enhancements to probing depth and attachment level (as compared to the results of using SRP alone).^29^^,^^30^ The current study demonstrated a noticeable impact of the aforementioned treatments on all studied groups. Specifically, the group receiving SRP plus the *S. persica* chip exhibited a greater decrease in PPD compared to the other groups. The PPD reduction (1.55 mm) in this group was more substantial compared to prior studies using SRP and other chips (such as chlorhexidine chips) on patients.^4^^,^^18^ The findings of this study were consistent with previous studies that used periodontal chips containing chlorhexidine as a local delivery agent to treat periodontitis.^22^^,^^29^^,^^30^ These previous studies demonstrated that when a biodegradable chlorhexidine chip is utilised as an adjunct to conventional periodontal therapy, it leads to critical enhancements in periodontal probing depth and attachment level compared to the results of using SRP alone. In addition, it was found that the clinical sign of periodontitis also significantly improved when a periodontal chip was used as an adjunct compared to SRP alone. The current study observed a significant gain in CAL in the group treated with *S. persica* chips compared to the other groups. This phenomenon could be attributed to the cumulative influence of the antibacterial properties of *S. persica* and chitosan (subjected to controlled release) or the effect of the synergistic interactions among the constituents of *S. persica*.

The current study utilised a 2.5 mg concentration of *S. persica* (which is consistent with the concentration found in chlorhexidine chips); in contrast, a dosage of 0.25 mg of BITC was employed. The authors of the current study stipulate that an increase in the concentration of BITC may yield more favourable outcomes. Nevertheless, the results of this study consistently showed post-treatment improvements in gingival inflammation, as evidenced by the significant changes in BOP before and after treatment in all groups. These findings align with the results from prior studies.^22^^,^^30^

Noticeable alterations in visible plaque were observed in all treatment groups that were administered chips, except for the control group that solely underwent SRP. These changes were observed both before and after the treatment.

This study was subject to certain limitations. It was not possible to compare *S. persica* chips with chlorhexidine chips due to financial constraints (the acquisition of chlorhexidine chips was deemed a costly prospect). Future studies on this topic should evaluate the efficacy of the *S. persica* chips through an extensive clinical trial, enhance the concentration of BITC and conduct a comparative analysis involving chlorhexidine chips that are currently available in the market. Moreover, periodontal chips containing *S. persica* can be used either during an appointment for SRP or during periodontal maintenance appointments.

## Conclusion

The utilisation of periodontal chips derived from *S. persica* and BITC, integrated into a chitosan base as a means of targeted drug delivery, offers clinical advantages in the treatment of periodontitis. These chips can be used as an adjunct to conventional SRP in the treatment of patients with periodontitis.

## Figures and Tables

**Table 1:** Mean of probing pocket depth pre- and post-treatment (N = 240; n = 60 for each group)

Treatment group	Mean PPD in mm	Mean PPD difference in mm	SD	T-statistic	*P* value
**Control**		1	0.567	11.152	=0.001
Pre-treatment	5.52				
Post-treatment	4.52				
**Chitosan**		0.81	0.759	10.204	=0.001
Pre-treatment	6.08				
Post-treatment	5.27				
***S. persica***		1.55	0.891	13.473	=0.001
Pre-treatment	7.15				
Post-treatment	5.60				
**BITC**		1.27	0.594	10.281	=0.001
Pre-treatment	5.90				
Post-treatment	4.63				

PPD = probing pocket depth; SD = standard deviation; BITC = benzyl isothiocyanate.
